# Association Between CT Attenuation and Mechanical Basket Lithotripsy Failure in Common Bile Duct Stones: A Single-Centre Retrospective Exploratory Study

**DOI:** 10.3390/jcm15176769

**Published:** 2026-08-31

**Authors:** Takafumi Tanimoto, Kohsaku Ohnishi, Yuki Ito, Yuhiko Katanosaka, Naoko Hayata, Akino Okamoto, Hiroki Murai, Tomohide Kurahashi, Shigeki Suemura, Motohiro Hirao, Takuya Yamada, Atsushi Hosui

**Affiliations:** Department of Gastroenterology and Hepatology, Osaka Rosai Hospital, 1179-3 Nagasone-cho, Kita-ku, Sakai 591-8025, Osaka, Japanhosui@osakah.johas.go.jp (A.H.)

**Keywords:** common bile duct stone, ERCP, mechanical lithotripsy, computed tomography, Hounsfield unit, exploratory study

## Abstract

**Background:** Mechanical lithotripsy is used for difficult common bile duct (CBD) stones, but failure to fragment a grasped stone can complicate endoscopic management. Evidence regarding the association between preprocedural computed tomography (CT) attenuation and mechanical lithotripsy outcome is limited. This study explored the association between CT attenuation of CBD stones and mechanical basket lithotripsy failure in a single-centre retrospective cohort. **Methods:** We retrospectively analysed 37 patients in whom a CBD stone was grasped with a basket catheter, could not be extracted directly, and underwent attempted intraductal mechanical crushing between 1 April 2017 and 31 March 2023. Lithotripsy failure was defined as inability to fragment the grasped stone despite use of a dedicated crusher handle. Continuous variables were compared using the Mann–Whitney U test and categorical variables using Fisher’s exact test. CT attenuation analyses were restricted to CT-positive stones with measurable attenuation values (*n* = 27). Exploratory receiver operating characteristic (ROC) analyses were performed, and areas under the curve (AUCs) were accompanied by nonparametric bootstrap 95% confidence intervals (CIs) based on 10,000 resamples. No clinical CT attenuation threshold was proposed. **Results:** Mechanical lithotripsy succeeded in 31 patients and failed in 6. CT attenuation was measurable in 27 patients (21 successes and 6 failures). Among these patients, the failure group had higher mean CT attenuation than the success group (median 241 [IQR 217.8–473.5] HU vs. 76 [67–88] HU; *p* = 0.00064) and higher maximum CT attenuation (386 [269.8–589.3] HU vs. 123 [102–133] HU; *p* = 0.00133). Exploratory ROC analysis showed an AUC of 0.968 (bootstrap 95% CI, 0.871–1.000) for mean CT attenuation and 0.940 (bootstrap 95% CI, 0.800–1.000) for maximum CT attenuation. These estimates were derived and evaluated in the same small dataset and were therefore considered exploratory. **Conclusions:** In this small, selected retrospective cohort, higher CT attenuation values were observed among the six patients with mechanical basket lithotripsy failure. These findings are exploratory and require confirmation in larger prospective studies using standardized CT protocols before CT attenuation can be incorporated into procedural decision-making.

## 1. Introduction

Common bile duct (CBD) stones are among the most common indications for therapeutic endoscopic retrograde cholangiopancreatography (ERCP), and endoscopic stone extraction is an established treatment for symptomatic choledocholithiasis. Following endoscopic sphincterotomy (EST), endoscopic papillary balloon dilation (EPBD), or endoscopic papillary large-balloon dilation (EPLBD), most CBD stones can be removed using conventional extraction balloons or basket catheters [1,2]. However, a subset of stones is considered difficult because of factors such as large stone size, stone impaction, distal bile duct narrowing, anatomical mismatch between the stone and distal bile duct, or surgically altered anatomy [1,3,4]. Such cases may require additional techniques, including mechanical lithotripsy, cholangioscopy-guided electrohydraulic lithotripsy (EHL), laser lithotripsy, or other rescue procedures [1,3,4,5].

Mechanical lithotripsy remains a useful treatment option when conventional stone extraction is unsuccessful. The procedure involves capturing a stone with a basket catheter and applying mechanical force to fragment it within the bile duct. Although mechanical lithotripsy is generally effective, fragmentation occasionally fails after the stone has been grasped. Such failure can complicate subsequent endoscopic management and may require additional papillary dilation, cholangioscopy-guided lithotripsy, or other rescue techniques [3,4,5,6,7].

Mechanical lithotripsy failure should be distinguished from basket impaction. Basket impaction is a procedural adverse event in which the basket and captured stone cannot be withdrawn or released, whereas the primary outcome evaluated in the present study was inability to mechanically fragment a stone that had already been grasped by a basket catheter. Basket impaction itself was not systematically evaluated as an outcome in this study.

Previous observational studies have identified several factors associated with difficult mechanical lithotripsy, including stone impaction, large stone diameter, and an unfavorable relationship between stone diameter and distal bile duct diameter [6,7]. These factors primarily describe anatomical or procedural difficulty and provide limited information regarding the intrinsic physical properties of the stone itself. Consequently, whether a stone can be mechanically fragmented after capture may remain difficult to anticipate before the procedure.

Stone composition may contribute to mechanical fragility. Calcium-containing biliary stones have physical and radiographic characteristics that differ from cholesterol-predominant stones. CT attenuation, expressed in Hounsfield units (HU), reflects radiographic density and may provide indirect information regarding stone composition. Previous CT studies of gallstones have shown associations between CT attenuation and gallstone composition or calcium content [8,9,10,11].

Abdominal CT is commonly performed during the clinical evaluation of patients with suspected or confirmed choledocholithiasis. Therefore, if routinely available CT attenuation measurements are associated with the ability to mechanically fragment CBD stones, they could provide useful preprocedural information without requiring an additional invasive examination. However, evidence regarding the association between CT attenuation and mechanical lithotripsy outcome in CBD stones remains limited.

Improved characterization of this association could contribute to hypothesis generation regarding procedural planning for difficult CBD stones. Nevertheless, because CT attenuation is affected by acquisition and reconstruction parameters and because previous evidence is limited, attenuation values should not be regarded as established clinical decision thresholds without appropriate validation.

Therefore, we conducted a single-centre retrospective exploratory association study to compare preprocedural CT attenuation and other clinical characteristics between patients with successful and unsuccessful mechanical basket lithotripsy. The primary objective was to explore the association between CT attenuation and mechanical lithotripsy failure rather than to develop or validate a prediction model or clinically applicable CT attenuation threshold.

## 2. Materials and Methods

### 2.1. Study Design and Participants

This was a single-centre retrospective exploratory association study conducted at Osaka Rosai Hospital. The manuscript was prepared with reference to the Strengthening the Reporting of Observational Studies in Epidemiology (STROBE) statement [12], and the completed STROBE Checklist is provided in the Supplementary Materials.

A total of 843 consecutive patients who underwent ERC/ERCP for CBD stones between 1 April 2017 and 31 March 2023 were retrospectively screened. Of these, 806 patients were excluded before formation of the analytic cohort: 250 because the target stone was not grasped with a basket catheter and 556 because the stone was extracted directly without requiring intraductal mechanical crushing. The remaining 37 patients, in whom a CBD stone was grasped with a basket catheter, could not be removed directly through the papilla, and subsequently underwent attempted intraductal mechanical crushing, constituted the final analytic cohort. Each patient contributed one ERCP procedure and one target stone to the analytic dataset; no patient contributed multiple procedures or multiple stones.

The study population therefore represented a selected subgroup of patients with difficult CBD stones requiring mechanical lithotripsy and should not be considered representative of all patients undergoing ERCP for choledocholithiasis.

No formal sample size calculation was performed because this retrospective study included all eligible patients who met the predefined inclusion criteria during the study period. The small number of mechanical lithotripsy failures (*n* = 6) limited statistical precision and the complexity of the analyses that could be reliably performed.

### 2.2. Outcome Definition and Endoscopic Procedure

The primary outcome was mechanical basket lithotripsy failure. When a stone could not be removed through the papilla after being grasped with a basket catheter, intraductal mechanical crushing was attempted. Lithotripsy failure was defined as inability to fragment the grasped stone despite use of a dedicated crusher handle.

This outcome was distinguished from basket impaction. Basket impaction itself was not systematically assessed as an outcome and was therefore not considered a predicted event in the present analysis.

The basket catheters used were Zemex crusher catheters (Zeon Medical Inc., Tokyo, Japan), including Tyco 3-wire/4-wire and Tyco small 6-wire devices, selected according to stone characteristics and procedural requirements. Preprocedural endoscopic papillary large-balloon dilation (EPLBD) was recorded as a procedural variable. Basket selection, papillary treatment, and the decision to continue or discontinue mechanical lithotripsy were made according to routine clinical judgment and were not governed by a prospectively standardized study protocol. The number of crushing attempts, operator experience, and explicit stopping criteria were not consistently available in the retrospective dataset and therefore could not be analyzed in a standardized manner.

### 2.3. CT Attenuation Measurement

All patients underwent multidetector computed tomography before ERCP as part of routine clinical evaluation. CT images were retrospectively reviewed using the institutional picture archiving and communication system (PACS).

For stones visible on CT, attenuation measurements were obtained on the axial image demonstrating the largest cross-sectional area of the target stone. A freehand region of interest (ROI) was manually placed to encompass the entire visible stone while avoiding adjacent bile, the biliary wall, and image artefacts as much as possible. Mean CT attenuation (HU) and maximum CT attenuation (HU) were recorded for each stone. In patients with multiple CBD stones, the target stone measured on CT was matched to the stone subjected to mechanical lithotripsy during ERCP by comparing stone diameter on preprocedural CT with stone diameter on fluoroscopic images obtained during ERCP; the stone with the corresponding diameter was selected for CT attenuation assessment.

Stones for which attenuation measurements could not be obtained because of insufficient CT visibility were classified as CT-negative/unmeasurable for attenuation analysis and were excluded from analyses involving CT attenuation values. These patients remained included in analyses of clinical characteristics. Of the 37 patients, CT attenuation was measurable in 27 (21 patients with successful lithotripsy and all 6 patients with lithotripsy failure).

CT attenuation measurements were performed retrospectively before statistical analysis by a gastroenterologist experienced in ERCP and biliary imaging who was also responsible for data collection. The assessor was not blinded to the mechanical lithotripsy outcome at the time of image review. Repeated measurements and formal assessment of intraobserver or interobserver reproducibility were not included in the original study protocol.

Further details of the CT attenuation measurement and statistical procedures are provided in the Supplementary Methods.

Detailed CT acquisition parameters, including scanner model, tube voltage, slice thickness, reconstruction algorithm, use of intravenous contrast, and the interval between CT and ERCP, were not available in the dataset used for the present retrospective analysis. CT examinations had been performed as part of routine clinical care rather than according to a prospectively standardized study protocol. This lack of standardized acquisition information was considered an important limitation when interpreting absolute HU values.

### 2.4. Statistical Analysis

Because only six mechanical lithotripsy failures occurred, no conventional multivariable logistic regression model was fitted, and no variable was interpreted as an independent predictor or independent risk factor.

Continuous variables were assessed descriptively and using the Shapiro–Wilk test. Because several distributions were non-normal and the failure group was small, between-group comparisons were performed using the Mann–Whitney U test. Continuous variables are reported as medians with interquartile ranges (IQRs). Median differences (failure minus success) are presented with nonparametric bootstrap 95% CIs based on 10,000 resamples as exploratory effect estimates.

Categorical variables were compared using Fisher’s exact test with exact denominators.

ROC analysis was restricted to the 27 patients with CT-positive/measurable stones and was considered strictly exploratory. AUCs were accompanied by nonparametric bootstrap 95% CIs based on 10,000 resamples. No CT attenuation cutoff was proposed or evaluated for clinical decision-making. Because the ROC analyses were conducted in the same small retrospective dataset without internal or external validation, the AUC estimates were interpreted cautiously and solely as exploratory measures of discrimination.

Two-sided *p* values < 0.05 were used descriptively without adjustment for multiple exploratory comparisons. Statistical reanalyses were performed using Python version 3.13.5 (Python Software Foundation, Beaverton, OR, USA),.with SciPy version 1.17.0 and scikit-learn version 1.8.0.

## 3. Results

### 3.1. Cohort and Participant Characteristics

**Figure 1:**
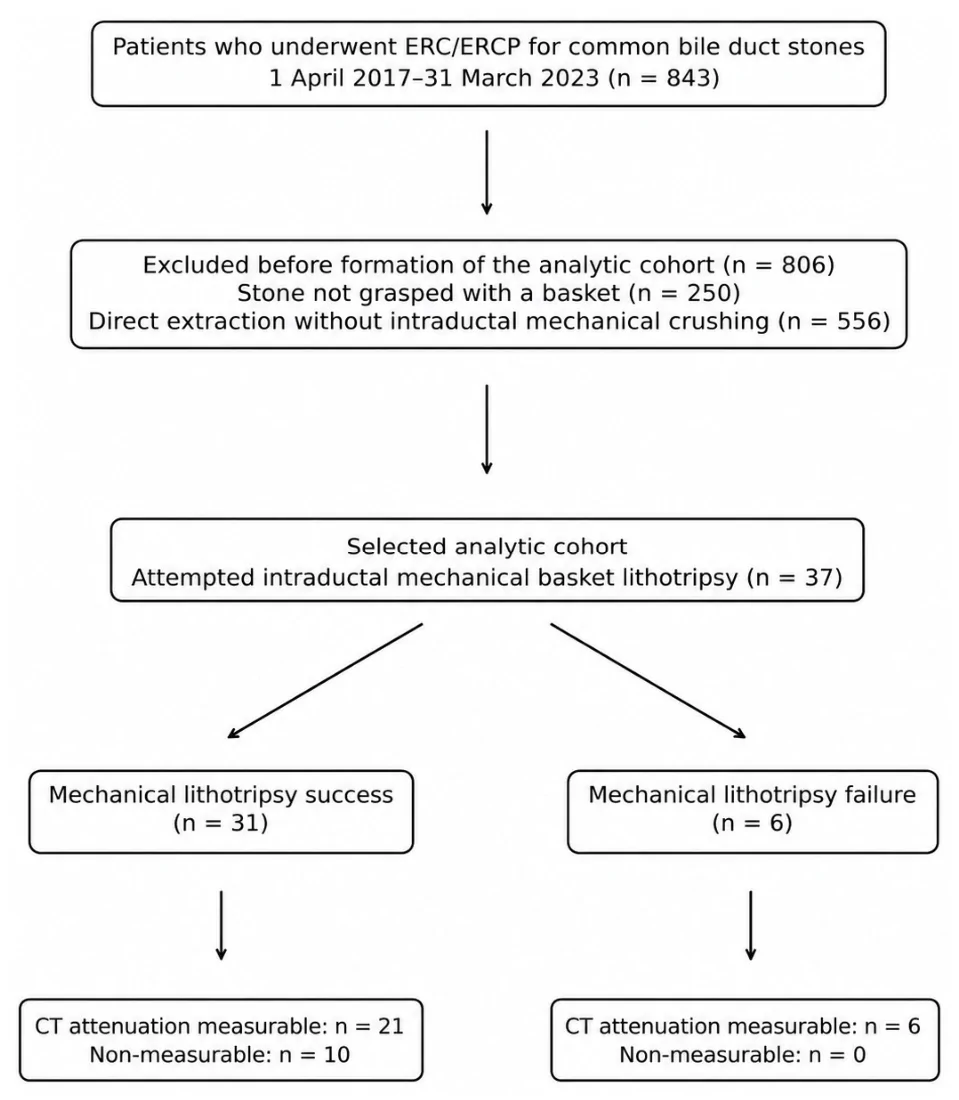
Participant flow diagram. Patients who underwent ERC/ERCP for common bile duct stones between 1 April 2017 and 31 March 2023 were screened (*n* = 843). A total of 806 patients were excluded before formation of the analytic cohort because the stone was not grasped with a basket (*n* = 250) or was extracted directly without intraductal mechanical crushing (*n* = 556). The selected analytic cohort comprised 37 patients undergoing attempted intraductal mechanical basket lithotripsy: 31 successes and 6 failures. CT attenuation was measurable in 27 patients (21 successes and 6 failures).

A total of 843 consecutive patients who underwent ERC/ERCP for common bile duct stones between 1 April 2017 and 31 March 2023 were retrospectively screened. Of these, 806 patients were excluded before formation of the analytic cohort: 250 because the target stone was not grasped with a basket catheter and 556 because the stone was extracted directly without requiring intraductal mechanical crushing. The remaining 37 patients, in whom a CBD stone was grasped with a basket catheter, could not be extracted directly, and underwent attempted intraductal mechanical lithotripsy, constituted the final analytic cohort (Figure 1). Mechanical basket lithotripsy was successful in 31 patients and unsuccessful in 6. There were 17 men and 20 women overall; the success group comprised 13 men and 18 women, whereas the failure group comprised 4 men and 2 women (Figure 1).

Patient and procedural characteristics of the success and failure groups are summarized in Table 1. The failure group was younger than the success group (median 66.5 vs. 81.0 years; *p* = 0.0036; median difference −14.5 years, bootstrap 95% CI −29.0 to −6.0). Stone diameter was smaller in the failure group (median 7.5 vs. 12.0 mm; *p* = 0.0327), whereas bile duct diameter did not differ materially between the groups (*p* = 0.836). The inverse association between stone diameter and lithotripsy failure was unexpected and was considered an unstable exploratory finding rather than evidence that smaller stones increase the risk of lithotripsy failure.

CT attenuation was measurable in 27 of the 37 patients (73.0%), including 21 of 31 patients in the success group and all 6 patients in the failure group. The remaining 10 patients had no measurable CT attenuation value and were excluded only from attenuation-based analyses.

Individual anonymized CT attenuation values for CT-positive/measurable stones are provided in Supplementary Table S1.

### 3.2. CT Attenuation Analysis

Among the 27 patients with CT-positive/measurable stones, mean CT attenuation was higher in the failure group than in the success group (median 241 [IQR 217.8–473.5] HU vs. 76 [67–88] HU; *p* = 0.00064). The median difference was 165 HU (bootstrap 95% CI, 81.5–569.5 HU).

Maximum CT attenuation showed a similar pattern. The median maximum attenuation was 386 [269.8–589.3] HU in the failure group and 123 [102–133] HU in the success group (*p* = 0.00133), corresponding to a median difference of 263 HU (bootstrap 95% CI, 61.0–790.0 HU).

The comparisons of mean and maximum CT attenuation between the success and failure groups are summarized in Table 2.

**Figure 2:**
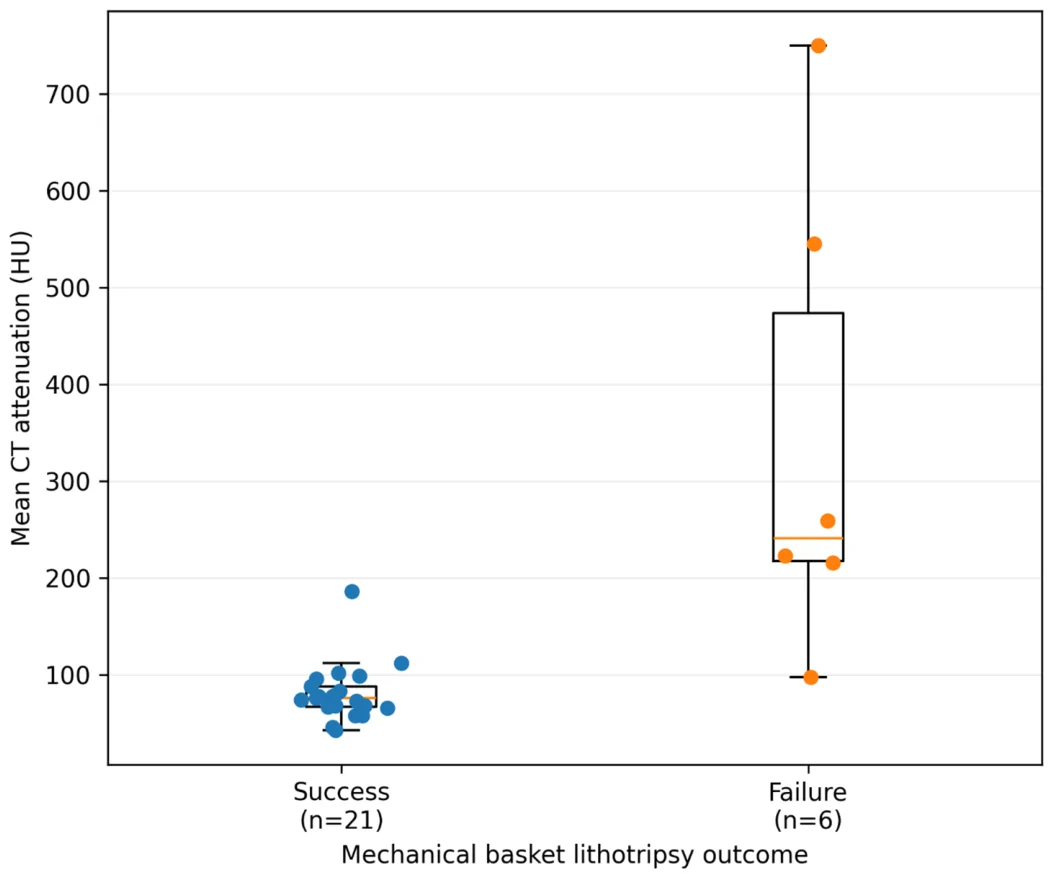
Distribution of mean CT attenuation according to mechanical basket lithotripsy outcome. Individual observations are shown together with box-and-whisker plots for CT-positive/measurable stones only (success, *n* = 21; failure, *n* = 6). Blue dots represent patients with successful mechanical lithotripsy, and orange dots represent patients with mechanical lithotripsy failure. Median mean CT attenuation was 76 [IQR 67–88] HU in the success group and 241 [217.8–473.5] HU in the failure group (Mann–Whitney U test, *p* = 0.00064).

**Figure 3:**
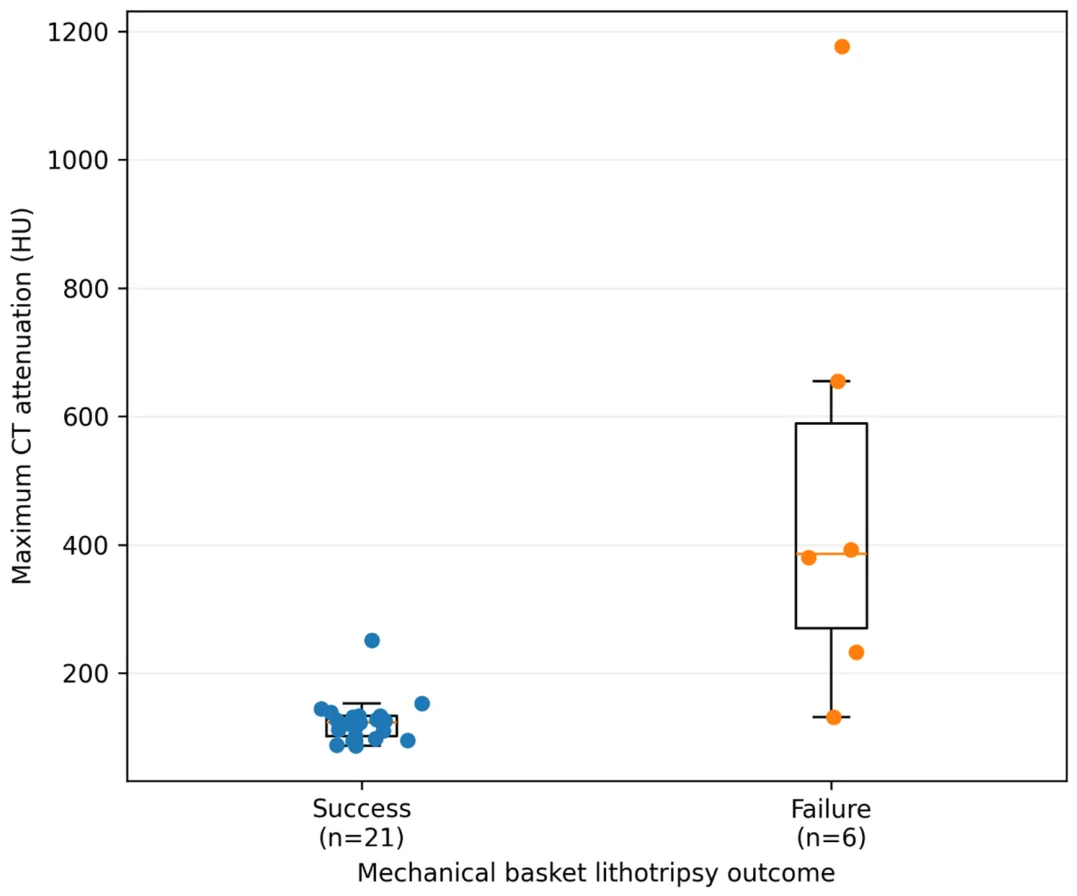
Distribution of maximum CT attenuation according to mechanical basket lithotripsy outcome. Individual observations are shown together with box-and-whisker plots for CT-positive/measurable stones only (success, *n* = 21; failure, *n* = 6). Blue dots represent patients with successful mechanical lithotripsy, and orange dots represent patients with mechanical lithotripsy failure. Median maximum CT attenuation was 123 [IQR 102–133] HU in the success group and 386 [269.8–589.3] HU in the failure group (Mann–Whitney U test, *p* = 0.00133).

Individual patient-level distributions of mean and maximum CT attenuation are shown in Figure 2 and Figure 3, respectively.

### 3.3. Exploratory ROC Analysis

Exploratory ROC analysis was performed in the 27 patients with measurable CT attenuation values. Mean CT attenuation yielded an AUC of 0.968 (bootstrap 95% CI, 0.871–1.000), whereas maximum CT attenuation yielded an AUC of 0.940 (bootstrap 95% CI, 0.800–1.000).

Detailed results of the exploratory ROC analysis, including bootstrap estimates, are provided in Supplementary Table S2.

**Figure 4:**
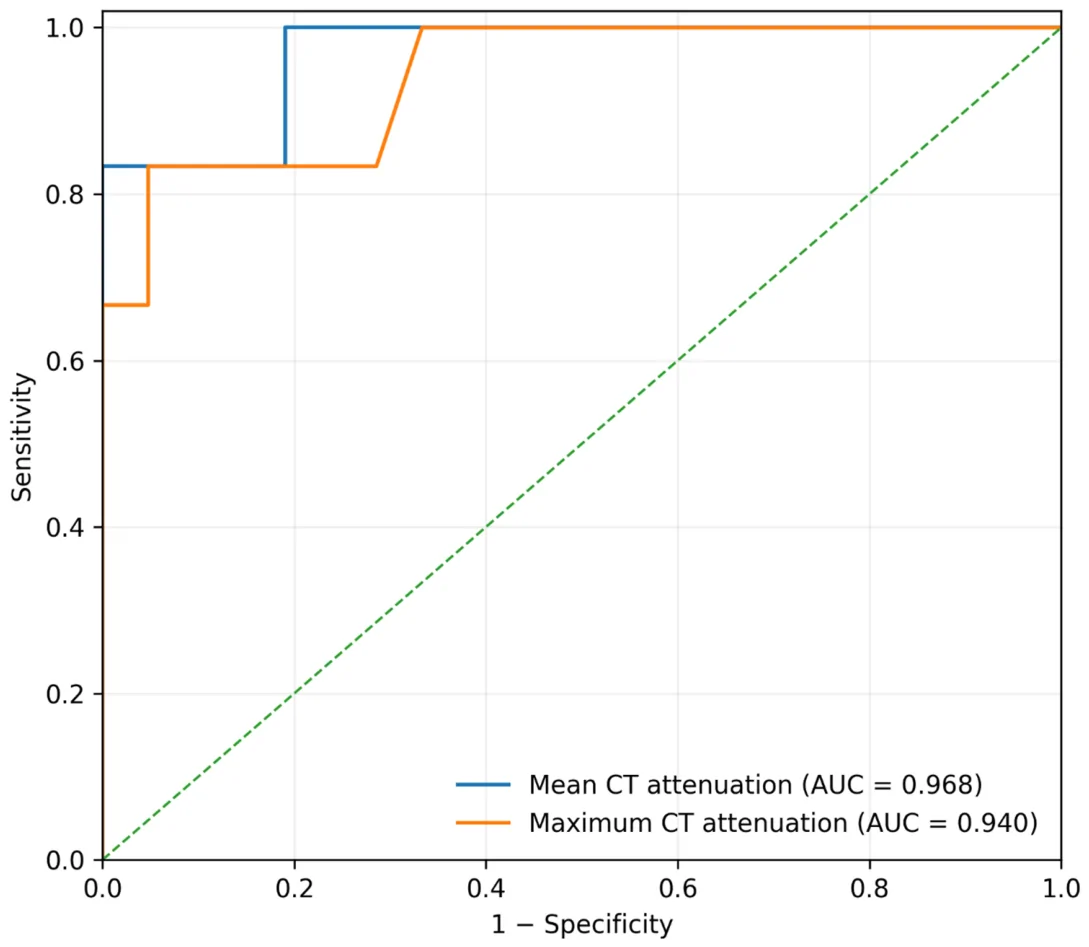
Exploratory ROC curves for mean and maximum CT attenuation. ROC analyses were restricted to the 27 patients with CT-positive/measurable stones. Mean CT attenuation yielded an AUC of 0.968 (nonparametric bootstrap 95% CI, 0.871–1.000), and maximum CT attenuation yielded an AUC of 0.940 (0.800–1.000). Bootstrap confidence intervals were based on 10,000 resamples. The analyses are exploratory, and no clinical CT attenuation cutoff is proposed.

These AUC estimates were derived and evaluated in the same small dataset containing only six lithotripsy failures and therefore are likely to be optimistic and unstable. No CT attenuation threshold was proposed, and the ROC findings should be regarded solely as exploratory (Figure 4).

### 3.4. Procedural Variables and Rescue Treatment

Basket use in the success group was 3-wire/4-wire/6-wire = 3/25/3, compared with 0/5/1 in the failure group. A 6-wire basket was used in 3 of 31 patients in the success group and 1 of 6 patients in the failure group (Fisher’s exact *p* = 0.524). Preprocedural EPLBD was recorded in 12 of 31 patients in the success group and 2 of 6 patients in the failure group (*p* = 1.000).

In the six patients with mechanical lithotripsy failure, emergency surgery was avoided. The stones were released or subsequently managed endoscopically, and additional endoscopic treatment, including papillary balloon dilation and/or EHL, enabled definitive stone management.

## 4. Discussion

In this single-centre retrospective exploratory study of a highly selected cohort of patients with difficult CBD stones requiring mechanical basket lithotripsy, higher CT attenuation values were observed among the six patients in whom mechanical fragmentation failed. Both mean and maximum CT attenuation were higher in the failure group than in the success group. Exploratory ROC analyses also demonstrated high apparent discrimination; however, these estimates were derived from only 27 CT-positive/measurable stones, including six failures, and should therefore be interpreted with considerable caution.

The present findings should be considered an association rather than evidence of a validated predictive relationship. In particular, the study was not designed to develop a clinical prediction model or establish a CT attenuation threshold for treatment selection. The small number of failure events precluded reliable multivariable modelling, and no variable should be interpreted as an independent predictor or independent risk factor on the basis of these data.

Several anatomical and procedural factors have previously been associated with difficult mechanical lithotripsy, including large or impacted stones and an unfavorable relationship between stone size and distal bile duct diameter [3,4]. In the present cohort, however, stone diameter was unexpectedly smaller in patients with lithotripsy failure. Given the small number of events, selected study population, and possible influence of basket selection and other procedural factors, this observation should be considered unstable and hypothesis-generating rather than evidence of a causal association.

A possible explanation for the observed association between CT attenuation and lithotripsy failure is variation in stone composition. Calcium-containing stones generally demonstrate higher CT attenuation, and differences in mineral composition could plausibly affect mechanical characteristics [8,9,10,11]. However, stone composition and physical hardness were not directly measured in this study. Therefore, the present data do not demonstrate that high-attenuation CBD stones are mechanically harder, and this interpretation remains a hypothesis requiring direct investigation.

The distinction between mechanical lithotripsy failure and basket impaction is also important. The primary outcome of this study was failure to fragment a grasped stone using a mechanical lithotriptor. Basket impaction was not systematically defined or assessed as a study outcome. Accordingly, the present results should not be interpreted as demonstrating that CT attenuation predicts basket impaction.

The exploratory ROC findings require particularly cautious interpretation. Although the observed AUCs were high, the analysis was restricted to 27 CT-positive/measurable stones and included only six failures. Moreover, discrimination was evaluated in the same dataset in which the association was identified, without internal or external validation. The apparent performance is therefore likely to be optimistic. For this reason, we do not propose a CT attenuation cutoff for clinical decision-making on the basis of the present study.

Nevertheless, the observed association may provide a rationale for further investigation. CT is frequently available before ERCP, and attenuation measurements can potentially be obtained from routinely acquired images without additional invasive procedures. If the association between CT attenuation and mechanical lithotripsy outcome is confirmed in larger, prospectively collected cohorts using standardized CT acquisition and measurement protocols, CT characteristics could potentially contribute to preprocedural characterization of difficult CBD stones. Such a role, however, remains to be established.

This study has several important limitations. First, it was a retrospective single-centre study with a small sample size and only six lithotripsy failures, resulting in substantial statistical uncertainty and precluding reliable multivariable modelling. Second, the cohort was highly selected and included only patients in whom a stone had already been grasped with a basket catheter, could not be directly extracted, and required attempted intraductal crushing. The results therefore cannot be generalized to all patients undergoing ERCP for CBD stones. Third, CT attenuation analyses were limited to the 27 patients with CT-positive/measurable stones; all six failures had measurable stones, whereas 10 patients in the success group did not, creating potential selection and spectrum bias. Fourth, CT examinations were obtained as part of routine clinical care rather than using a prospectively standardized acquisition protocol, and detailed acquisition and reconstruction parameters were unavailable for the present analysis. Absolute HU values may therefore have been influenced by differences in CT technique. Fifth, CT attenuation measurements were performed retrospectively by a gastroenterologist experienced in ERCP and biliary imaging who was also responsible for data collection and was aware of the mechanical lithotripsy outcome at the time of image review; measurement bias therefore cannot be excluded. Formal intraobserver and interobserver reproducibility assessments were not performed. Sixth, stone composition and mechanical hardness were not directly measured. Seventh, procedural factors, including basket selection, papillary treatment, operator-related factors, and rescue strategies, were not fully standardized and may have contributed to residual confounding and confounding by indication. In addition, the number of crushing attempts, operator experience, and explicit criteria for discontinuing mechanical lithotripsy were not consistently available in the retrospective dataset, limiting standardized assessment of procedural decision-making. Finally, the ROC analysis was performed in the same small dataset without internal or external validation, and the resulting AUC estimates should therefore be regarded as exploratory and potentially optimistic.

Future studies should prospectively enroll larger and more representative populations, standardize CT acquisition and ROI measurement protocols, evaluate intraobserver and interobserver reproducibility, and directly assess stone composition and physical characteristics where feasible. Independent validation will be essential before CT attenuation can be used to inform procedural decision-making.

## 5. Conclusions

In this small, selected retrospective cohort, higher CT attenuation values were observed among the six patients with mechanical basket lithotripsy failure. These findings represent an exploratory association and do not establish a predictive model or clinically applicable CT attenuation threshold. Confirmation in larger prospective studies using standardized CT acquisition and measurement protocols and independent validation is required before CT attenuation can be incorporated into procedural decision-making.

## Figures and Tables

**Table 1:** Patient and procedural characteristics according to mechanical lithotripsy outcome.

Variable	Success (*n* = 31)	Failure (*n* = 6)	*p* Value
Age, years	81 [74–85]	66.5 [60.75–67]	0.0036
Male sex, *n*/*N* (%)	13/31 (41.9)	4/6 (66.7)	0.383
Postoperative reconstructed intestine, *n*/*N* (%)	4/31 (12.9)	1/6 (16.7)	1.000
Stone diameter, mm	12 [10–17.5]	7.5 [5.5–11.75]	0.0327
Bile duct diameter, mm	11 [8–17]	10.5 [9.25–11]	0.836
Preprocedural EPLBD, *n*/*N* (%)	12/31 (38.7)	2/6 (33.3)	1.000
6-wire basket, *n*/*N* (%)	3/31 (9.7)	1/6 (16.7)	0.524
CT attenuation measurable, *n*/*N* (%)	21/31 (67.7)	6/6 (100)	0.162

Data are median [interquartile range] unless otherwise indicated. Continuous variables were compared using the Mann–Whitney U test; categorical variables were compared using Fisher’s exact test. EPLBD, endoscopic papillary large-balloon dilation; CT, computed tomography.

**Table 2:** CT attenuation according to mechanical lithotripsy outcome among CT-positive/measurable stones.

Variable	Success (*n* = 21)	Failure (*n* = 6)	Median Difference (Bootstrap 95% CI)	*p* Value
Mean CT attenuation, HU	76 [67–88]	241 [217.8–473.5]	165 (81.5–569.5)	0.00064
Maximum CT attenuation, HU	123 [102–133]	386 [269.8–589.3]	263 (61.0–790.0)	0.00133

CT attenuation analyses were restricted to 27 patients with CT-positive/measurable stones. Median differences are failure minus success. Bootstrap 95% confidence intervals were based on 10,000 nonparametric resamples. No CT attenuation threshold is proposed.

## Data Availability

The data are not publicly available because they contain information that could compromise the privacy of research participants. De-identified data are available from the corresponding author upon reasonable request and with approval from the institutional ethics committee.

## Supplementary Materials

The following supporting information can be downloaded at: https://www.mdpi.com/article/10.3390/jcm15176769/s1, Supplementary Table S1, Individual anonymized CT attenuation values for CT-positive/measurable stones; Supplementary Table S2, Exploratory ROC analysis with bootstrap estimates; Supplementary Methods, Detailed CT attenuation measurement and statistical procedure; Supplementary File S1, STROBE Statement—Checklist of items that should be included in reports of observational studies. The participant flow diagram is presented exclusively as Figure 1 in the main manuscript.
