# Supplementary material for: Association Between CT Attenuation and Mechanical Basket Lithotripsy Failure in Common Bile Duct Stones: A Single-Centre Retrospective Exploratory Study
Source: J Clin Med. 2026 Aug 31;15(17):6769. doi: 10.3390/jcm15176769 (PMC13566551; doi:10.3390/jcm15176769)
Supplement: Supplementary file 1 [file jcm-15-06769-s001.zip › jcm-4436653-supplementary.pdf]

Supplementary Materials

Association Between CT Attenuation and Mechanical Basket Lithotripsy Failure in Common Bile Duct Stones: A Single-Centre Retrospective Exploratory Study

Supplementary Table S1. Individual anonymized CT attenuation values for CT-positive/measurable stones

CT attenuation was measurable in 27 patients: 21 with successful mechanical lithotripsy and 6 with failure. Study IDs below are newly generated and do not correspond to patient identifiers.

Study ID	Mechanical lithotripsy outcome	Mean CT attenuation (HU)	Maximum CT attenuation (HU)
CT-01	Success	186	251
CT-02	Success	88	139
CT-03	Success	102	133
CT-04	Success	78	112
CT-05	Failure	98	131
CT-06	Success	46	94
CT-07	Success	78	131
CT-08	Success	96	127
CT-09	Success	67	121
CT-10	Success	43	87
CT-11	Success	112	153
CT-12	Success	58	98
CT-13	Success	74	144
CT-14	Failure	259	392
CT-15	Success	68	117
CT-16	Success	66	95
CT-17	Success	79	102
CT-18	Failure	223	380
CT-19	Failure	216	233
CT-20	Success	76	88
CT-21	Success	58	110
CT-22	Success	99	133
CT-23	Success	68	126
CT-24	Success	83	123
CT-25	Failure	750	1177
CT-26	Success	73	128
CT-27	Failure	545	655

Supplementary Table S2. Exploratory ROC analysis with bootstrap estimates

ROC analyses were restricted to the 27 patients with CT-positive/measurable stones and were considered strictly exploratory. No CT attenuation cutoff, sensitivity, or specificity is proposed for clinical decision-making. Nonparametric bootstrap confidence intervals were based on 10,000 resamples.

CT attenuation measure	n	Failures	AUC	Nonparametric bootstrap 95% CI	Bootstrap resamples
Mean CT attenuation	27	6	0.968	0.871–1.000	10,000
Maximum CT attenuation	27	6	0.940	0.800–1.000	10,000

Supplementary Methods. Detailed CT attenuation measurement and statistical procedure

CT image review and attenuation measurement

All patients underwent multidetector computed tomography before ERCP as part of routine clinical evaluation. CT images were retrospectively reviewed using the institutional picture archiving and communication system (PACS). For stones visible on CT, attenuation measurements were obtained on the axial image demonstrating the largest cross-sectional area of the target stone. A freehand region of interest (ROI) was manually placed to encompass the entire visible stone while avoiding adjacent bile, the biliary wall, and image artefacts as much as possible. Mean CT attenuation and maximum CT attenuation were recorded in Hounsfield units (HU).

Handling of CT-negative/non-measurable stones

Stones for which attenuation measurements could not be obtained because of insufficient CT visibility were classified as CT-negative/non-measurable for attenuation analysis. These patients were excluded only from analyses involving CT attenuation values and remained included in analyses of clinical characteristics. Of 37 patients in the analytic cohort, CT attenuation was measurable in 27 (21 with successful lithotripsy and all 6 with lithotripsy failure).

Measurement reproducibility

CT attenuation measurements were performed retrospectively before statistical analysis. The original study protocol did not include formal assessment of intraobserver or interobserver reproducibility. This limitation should be considered when interpreting the attenuation measurements.

CT acquisition parameters

Detailed CT acquisition parameters, including scanner model, tube voltage, slice thickness, reconstruction algorithm, use of intravenous contrast, and the interval between CT and ERCP, were not available in the dataset used for the present retrospective analysis. CT examinations had been performed as part of routine clinical care rather than according to a prospectively standardized study protocol. This lack of standardized acquisition information was considered an important limitation when interpreting absolute HU values.

Exploratory statistical analysis

Continuous variables were compared using the Mann–Whitney U test and categorical variables using Fisher's exact test. Because only six mechanical lithotripsy failures occurred, no conventional multivariable logistic regression model was fitted and no variable was interpreted as an independent predictor or independent risk factor. Median differences and exploratory AUC estimates were accompanied by nonparametric bootstrap 95% confidence intervals based on 10,000 resamples. ROC analyses were restricted to the 27 patients with CT-positive/measurable stones. No clinically applicable CT attenuation threshold was proposed.

Supplementary File S1. STROBE Statement—Checklist of items that should be included in reports of observational studies

Checklist adapted for a cohort-type observational report. Locations refer to sections of the revised manuscript rather than fixed page numbers so that the checklist remains valid after final typesetting.

Item	STROBE recommendation	Location in revised manuscript
1	Indicate the study design with a commonly used term in the title or abstract; provide an informative and balanced abstract.	Title; Abstract
2	Explain the scientific background and rationale for the investigation.	Introduction
3	State specific objectives, including any prespecified hypotheses.	Introduction, final paragraph
4	Present key elements of study design early in the paper.	Methods 2.1
5	Describe the setting, locations, and relevant dates, including periods of recruitment/exposure/follow-up/data collection.	Methods 2.1; study period
6	Give eligibility criteria and the sources and methods of participant selection.	Methods 2.1; Figure 1 (main manuscript)
7	Clearly define outcomes, exposures, predictors, potential confounders, and effect modifiers.	Methods 2.2–2.4
8	For each variable of interest, give sources of data and details of methods of assessment.	Methods 2.3; Supplementary Methods
9	Describe efforts to address potential sources of bias.	Methods; Discussion, Limitations
10	Explain how the study size was arrived at.	Methods 2.1; all eligible cases in selected analytic cohort
11	Explain how quantitative variables were handled in the analyses.	Methods 2.4
12	Describe all statistical methods, methods used to examine subgroups/interactions, handling of missing data, and sensitivity analyses where applicable.	Methods 2.4; Supplementary Methods
13	Report numbers of individuals at each stage of the study and reasons for non-participation/exclusion.	Results 3.1; Figure 1 (main manuscript)
14	Give characteristics of study participants and information on exposures and potential confounders; indicate missing data.	Results 3.1; main tables
15	Report numbers of outcome events or summary measures over time.	Results 3.1
16	Give unadjusted estimates and their precision; make clear which confounders were adjusted for and why.	Results 3.2–3.3; no multivariable adjusted model fitted
17	Report other analyses done, including subgroup/sensitivity analyses where applicable.	Results 3.3; exploratory ROC analysis
18	Summarize key results with reference to study objectives.	Discussion, opening paragraph
19	Discuss limitations, taking into account sources of potential bias or imprecision.	Discussion, Limitations
20	Give a cautious overall interpretation considering objectives, limitations, multiplicity, and other evidence.	Discussion; Conclusions
21	Discuss the generalisability (external validity) of the study results.	Discussion, Limitations
22	Give the source of funding and role of funders.	Back Matter: Funding
